# Local and Systemic STAT3 and p65 NF-KappaB Expression as Progression Markers and Functional Targets for Patients With Cervical Cancer

**DOI:** 10.3389/fonc.2020.587132

**Published:** 2020-11-19

**Authors:** Renata A. M. Rossetti, Ildefonso A. da Silva-Junior, Gretel R. Rodríguez, Karla L. F. Alvarez, Simone C. Stone, Marcella Cipelli, Caio R. F. Silveira, Mariana Carmezim Beldi, Giana R. Mota, Paulo F. R. Margarido, Edmund C. Baracat, Miyuki Uno, Luisa L. Villa, Jesus P. Carvalho, Kaori Yokochi, Maria Beatriz S. F. Rosa, Noely P. Lorenzi, Ana Paula Lepique

**Affiliations:** ^1^ Department of Immunology, Instituto de Ciências Biomédicas, Universidade de Sao Paulo, São Paulo, Brazil; ^2^ Department of Radiology and Oncology, Faculdade de Medicina da Universidade de Sao Paulo, Instituto do Câncer do Estado de São Paulo, São Paulo, Brazil; ^3^ Hospital Universtário, Universidade de Sao Paulo, São Paulo, Brazil; ^4^ Biobanco da Rede Acadêmica de Pesquisa do Câncer da Universidade de Sao Paulo, São Paulo, Brazil; ^5^ Center for Translational Research in Oncology, Instituto do Câncer do Estado de São Paulo, São Paulo, Brazil

**Keywords:** cervical cancer, human papillomavirus, immunomodulation, immunoevasion, nuclear factor – kappa B, signal transducer activator of transcription 3

## Abstract

Cervical cancer, which main etiologic factor is Human Papillomavirus (HPV) infection, continues to be a burden for public health systems in developing countries. Our laboratory has been working with the hypothesis that signals generated in the tumor microenvironment can modulate local and systemic immune responses. In this context, it would be reasonable to think that tumors create pro-tumoral bias in immune cells, even before they are recruited to the tumor microenvironment. To understand if and how signaling started in the tumor microenvironment can influence cells within the tumor and systemically, we investigated the expression of key proteins in signaling pathways important for cell proliferation, viability, immune responses and tolerance. Besides, we used detection of specific phosphorylated residues, which are indicative of activation for Akt, CREB, p65 NFκB, and STAT3. Our findings included the observation of a significant STAT3 expression increase and p65 NFκB decrease in circulating leukocytes in correlation with lesion grade. In light of those observations, we started investigating the result of the inhibition of STAT3 in a tumor experimental model. STAT3 inhibition impaired tumor growth, increased anti-tumor T cell responses and decreased the accumulation of myeloid cells in the spleen. The concomitant inhibition of NFκB partially reversed these effects. This study indicates that STAT3 and NFκB are involved in immunomodulatory tumor effects and STAT3 inhibition could be considered as therapy for patients with cervical cancer.

## Introduction

Cervical cancer continues to be a public health system problem in several countries worldwide (1). Persistent infection with high oncogenic risk Human Papillomavirus (HPV) is the main etiologic factor for cervical and other anogenital and oropharyngeal cancers (2, 3). In spite of the efficient prophylactic vaccines available, there is still demand for therapeutic tools against HPV associated cancers.

Signals generated in the tumor microenvironment may modulate immune cellular responses locally as well as systemically. HPV transformed cells secrete a variety of molecules such as IL-6, IL-8, G-CSF, TGFβ, lactate, PGE2 and, others (4–6). These factors can signal through receptors coupled to major intracellular pathways that regulate the tumor cell phenotype and immune responses, helping to determine tumor fate. For example, through NFκB activity, cervical cancer cells secrete IL-6, which in turn activates STAT3 with a pro-tumoral effect (7). However, IL-6 together with PGE2 can promote tolerogenic phenotype on antigen-presenting cells, therefore, facilitating tumor growth (5). Moreover, signaling triggered by G-CSF, also expressed by cervical cancer cells, activates STAT3 and has been shown to promote the accumulation of tolerogenic myeloid cells facilitating tumor growth (6, 8). Data obtained from experimental HPV-associated tumor models also showed that these tumors cause several effects on the immune system including triggering of regulatory T cell responses and accumulation of myeloid-derived suppressor cells in secondary lymphoid organs (4, 9).

Based on the premise that tumors can systemically signal to modulate immune responses, which may lead to recruitment of pro-tumoral biased leukocytes to the tumor microenvironment, our laboratory decided to investigate the expression and potential activation status of proteins involved in major intracellular signaling pathways involved in cell proliferation, inflammation, immune responses, and tolerance. Moreover, we investigated the expression of these protein s in cervical biopsies and peripheral blood leukocytes from patients with high-grade cervical lesions and invasive cancer. Our chosen targets were STAT3, p65 NFκB, Akt, and CREB. Cells tightly control the expression and activity of these proteins. A key event in these proteins’ activity control is phosphorylation. We took advantage of commercial antibodies that recognize phosphorylated residues that are indicative of activation so we could not only investigate the protein expression but also detect potential activation of each of the indicated pathways (10–13). STAT3 is considered an oncogene due to its activity regulating transcription and metabolism (14). It is involved in inflammatory responses, in the differentiation of CD4 T lymphocytes, in epithelial-mesenchymal transitions, cell proliferation, and tolerance to tumor antigens (15). NFκB comprises a family of transcription factors involved in inflammation and immune responses, as well as cancer cell proliferation and survival (16). The PI3K/Akt pathway controls cell survival, proliferation, and metabolism, and is essential for lymphocyte activation (17). Finally, we also investigated CREB activation, which through cAMP-responsive element-binding protein is a transcription factor that can be activated by the PI3K/Akt pathway. CREB controls cell metabolism and is involved in tolerogenic and anti-inflammatory responses (18).

We were able to show that all these proteins display a basal expression level in biopsies, with little variation in expression depending on lesion grade. In the peripheral blood, however there was an increase in STAT3 expression and a decrease in p65 NFκB expression in the circulating leukocytes dependent on lesion grade. Moreover, we were able to perform Pearson correlation with the protein expression data and could show that blood cells from cancer patients have a different correlation profile than cells from clinically healthy donors. We also studied an HPV associated experimental model to better understand the potential effects of blocking STAT3, one of the signaling proteins investigated, on tumor growth and anti-tumor immune responses. Finally, here we describe how tumor cells co-opt signaling pathways in the tumor microenvironment and systemically to promote immune suppression, tumor growth, and progression. Our results suggest that inhibiting STAT3 may be a useful tool for cervical cancer or high-grade lesions treatment.

## Materials and Methods

### Patients’ Study Design

This study was approved by the Institutional Review Boards from Instituto de Ciências Biomédicas, Hospital Universitário and Instituto do Câncer do Estado de São Paulo, Comissão Nacional de Ética em Pesquisa process number 02083912.6.0000.5467. All patients and control subjects signed an informed consent form before sample harvesting. All methods followed the guidelines established by the Brazilian National Committee in Ethical Research. Patients with diagnosis of high-grade lesions or cervical cancer were enrolled at Hospital Universitário or Instituto do Câncer do Estado de São Paulo at the moment of the first treatment. For the patients’ cohort, the inclusion criterion was the diagnosis of a high-grade lesion or cervical cancer. The exclusion criteria were immunosuppression or immunodeficiency, previous treatment for cervical disease, other treatments that could change the systemic status of the signaling pathways that were investigated, previous cervical biopsy in less than a month, and age below 18 years old. For control subjects, exclusion criteria were immunosuppression or immunodeficiency, any type of cancer diagnosis, treatments that could change the systemic status of the signaling pathways that were investigated, and age below 18 years old. We collected blood and cervical biopsy from the patients and only blood from the control subjects. After harvesting, biopsies were immediately transferred to a conical tube containing 5 ml of sterile RPMI 1640 (Thermo Fisher Scientific, Carlsbad, CA). Peripheral blood was harvested to a Vacutainer tube containing EDTA to prevent coagulation (Becton-Dickinson, Sunnyvale, CA). We harvested approximately 10 ml of blood per patient or control subject. Both biopsies and blood samples were processed within 2 h after harvesting. We enrolled 26 patients with CIN3 (cervical intraepithelial neoplasia 3), 20 with squamous cell carcinoma, 7 with adenocarcinoma, and 11 clinically healthy donors. From these samples, we were able to obtain data from 17 biopsies from high-grade lesion bearing patients, 15 from patients with squamous cell carcinoma, and 6 from patients with adenocarcinoma. The reason for this was the low yield of cells in some of the biopsies. In many cases, we had to prioritize one of two signaling pathways because we did not have enough cells for labeling with the antibodies to all signaling proteins. Our priority list was NFκB, STAT3, Akt, and CREB based on: 1) we had previously shown that in experimental models NFκB was linked to anti-tumor immune responses (19); 2) we had previously shown that patients with cancer had higher plasma concentration of G-CSF than controls and this cytokine activates STAT3; 3) data in the literature also indicate that STAT3 is involved in the carcinogenic process induced by HPV (8, 20, 21); finally, the Akt pathway is involved in tumor progression and metabolic changes in tumor cells (17). Therefore, the data presented in our work is not uniform regarding the number of samples for each signaling pathway. Each figure legend specifies the number of samples analyzed per experiment.

### Biopsy Processing and Flow Cytometry Analyses

Biopsies were digested with Collagenase I and IV (Worthington Biochemical Corp. Lakewood, NJ) and filtered to obtain a single-cell suspension. This method is very efficient to release leukocytes from biopsies, but not so efficient to release epithelial cells, without decreasing cell viability. Therefore, the percentages of leukocytes in biopsies will seem higher than expected. Single-cell suspensions were then labeled with anti-CD3 (clone HIT3a), anti-HLA-DR (clone G46-6), and anti-CD45 (clone HI30) (BD Biosciences, San Jose, CA). For the intracellular staining, first cells were fixed with 3.7% buffered formaldehyde, washed with phosphate-buffered saline (PBS), and then permeabilized with ice-cold 90% methanol at −20°C for 10 min and washed with PBS again (22). Cells were split into tubes to be incubated with rabbit polyclonal antibodies against the signaling proteins: phosphorylated-Akt (Thr308), total Akt, phosphorylated-p65 NFκB (Ser536), total p65 NFκB, phosphorylated-STAT3 (Tyr705), total Akt, phosphorylated-CREB (Ser133), total CREB (Cell Signaling Technology, Danvers, MA). Detection of the signaling proteins was through labeling with secondary anti-rabbit antibody conjugated with Alexa 488 or Alexa 647 (Cell Signaling Technology, Danvers, MA). Cells were analyzed in a FACSCanto (BD Biosciences, San Jose, CA), where at least 10,000 events per sample were acquired. We tested each antibody using HeLa cells, which are positive to all investigated markers (**Supplementary Figure 1**). We also saved a fragment of each biopsy for DNA extraction and HPV genotyping as described below.

### HPV Genotyping

An aliquot of 150 ng of phenol/chloroform purified DNA from each cervical biopsy was used for PCR amplification with PGMY09/11 primers (23). The amplicons were hybridized with probes from the LinearArray HPV genotyping test (Roche Molecular Diagnostics, Alameda, CA) for HPV genotyping.

### Peripheral Blood Processing and Flow Cytometry Analyses

Peripheral blood mononuclear cells (PBMCs) were isolated by Ficoll-Paque PLUS density gradient (GE Health Care Life Sciences, UK). Cell suspensions were treated similarly as described for biopsies, except for labeling with anti-CD45 antibody. Also, flow cytometry acquisition was at least 10,000 events.

### Murine Tumor Model

All studies in mice were approved by the Animal Ethics Committee at the Instituto de Ciências Biomédicas, process number 5/2015-E, and followed the guidelines established by the National Council for Control of Animal Experimentation. Five to 7 weeks old C57Black/6 female mice were inoculated with 10^5^ TC-1 tumor cells, a murine tumor cell line that expresses HPV16 oncogenes (24), in 100 µl of 0.5 mM MgCl_2_, 1mM CaCl_2_ supplemented PBS subcutaneously on the right flank. Cell viability for injection was always above 95%. Mice were observed daily until tumors were palpable, approximately 2 mm diameter, achieved at 7 to 10 days post tumor cell inoculation. Once tumors were palpable, mice were randomized into groups and treated with neutralizing antibodies, purified rat IgG anti-IL-6 or anti-G-CSF or the combination of both every other day through intraperitoneal injections in a final volume of 100 µl PBS/mouse. As antibody treatment control group, we used an injection of irrelevant rat IgG isotype. Alternatively, mice were treated with 5 mg/Kg STAT3 inhibitor (NSC74859) alone or together with 3 mg/Kg p65 NFκB inhibitor (JSH-23) every day for 7 days (Tocris Bioscience, Minneapolis, MN). Both inhibitors were diluted in 2% DMSO/PBS for injection, therefore, tumor-bearing control mice were treated with the same solution minus inhibitors. Mice were euthanized before tumors reached 1cm maximum diameter, or even before that to maintain the observed differences between controls and treated mice. We harvested spleens, tumors, and lymph nodes from each mouse. Cell suspensions were prepared as previously described (19). Tumor and spleen cell suspensions were used for immunophenotyping. Tumor and spleen fragments were also snap-frozen for immunofluorescence detection of STAT3 and NFκB. Immunofluorescence was performed on 5 µm cryosections incubated with rabbit anti-phosphorylated STAT3 or anti-phosphorylated p65 NFκB. Detection was performed with Alexa 488 conjugated anti-rabbit antibody. Tissues were then mounted with DAPI containing Fluorshield (Sigma Aldrich, San Luis, MO). Images were acquired using a BX61 fluorescence microscope and a DP70 camera and software (Olympus, JP).

The lymph node cell suspensions were used for the lymphocyte chimeras and T cell proliferation analysis. For the lymphocyte chimeras, cells obtained from the peripheral lymph nodes were counted, washed, sedimented, and resuspended in 0.5 mM MgCl_2_, 1mM CaCl_2_ supplemented PBS, and then injected into TC-1 tumor-bearing RAG1-/- mice. At the end of the experiment, tumors and lymph nodes were harvested for immunophenotyping. For flow cytometry we used the following antibodies: anti-CD45 (clone 30-F11), anti-CD4 (clone RM4-5), anti-CD8 (clone 53-6.7), anti-CD11b (clone M1/70), anti-Ly6C (clone AL-21), anti-Ly6G (clone 1A8). Antibodies were purchased from BD Biosciences (Carlsbad, CA), BioLegend (San Diego, CA), or R&D Systems (Minneapolis, MN). For the signaling proteins staining, we used the same antibodies as described before.

For the T cell proliferation assays lymph node suspensions were labeled with a violet proliferation dye (BD Biosciences, Carlsbad, CA) before seeding 2x10^5^ cells/well of a U bottom cell culture plate. Cells were left untreated or treated with 5 µg/ml HPV16 E6 and E7 peptides (25, 26), or treated with 1 µg/ml ionomycin and 10 ng/ml TPA (Phorbol-12-myristate-13-acetate - Cell Signaling Technology, Danvers, MA). At the end of 4 days incubation, cells were labeled with anti-CD4 and anti-CD8 for flow cytometry analysis, using a FACSCanto (BD Biosciences, Carlsbad, CA), where at least 30,000 events were acquired per sample.

### Data Analysis

Cell frequency data from both human and mouse experiments were compared by ANOVA. Protein expression from flow cytometry assays was represented as median fluorescence intensity (MFI) and the values (subtracting the MFI signal from unstained controls) were plotted on boxplots. For studies comparing protein expression levels with disease grade, data were compared by ANOVA. Pearson correlations were also performed to compare the expression of one given protein against all others in the same sample. The R values obtained were plotted as heatmaps, where * indicated that the positive or negative correlation had statistical significance. To increase statistical power, we have pooled together data from both cervical cancer histologic types. However, it is important to bear in mind that there are differences between squamous cell and adenocarcinoma of the uterine cervix. Notably, they have different origins, ecto and endocervix, respectively and they also have molecular differences (25). Nevertheless, squamous cell carcinomas and adenocarcinomas have many similarities, including the expression of HPV oncoproteins on tumor cells and the tumor microenvironment. In the experimental models, tumor growth kinetics were compared using the Mann Whitney U test. T cell proliferation and cytokine secretion data was also tested by ANOVA. In all cases, p values ≤ 0.05 were accepted to confirm that differences between results were significant.

## Results

### Cervical Cancer Patients Display Alterations in the Expression of Signaling Proteins In Circulating Leukocytes

A significant percentage of cervical intraepithelial neoplasia 3 (CIN3) progress to cervical cancer (26). Tumors originated from the ectocervix cells are squamous cell carcinomas (SCC) and the ones that originated from the endocervix are adenocarcinomas (AdC). Besides the histologic origin difference, patients with AdC usually have a poorer prognosis and there is discussion in the literature about the value of different treatments to each type of cancer (27, 28). We have studied a cohort of 53 patients with CIN3, SCC, and AdC (**Table 1**). Patients were enrolled either at the Hospital Universitário or at Instituto do Câncer do Estado de São Paulo, both at the metropolitan area of the city of São Paulo. From the patients, we harvested a biopsy and peripheral blood before treatment or surgery. The majority of cancer patients had grade I stage, according to the International Federation of Gynecology and Obstetrics (FIGO) classification. Our patients displayed a similar lesion profile to what has been described in other studies: 74% of the cancer patients had SCC and 26% AdC. Regarding HPV infection, as expected, only high oncogenic risk types were detected in CIN3 and cancer samples. HPV16 genotype was the most frequent (32%), followed by HPV18 (15%) and 26% of other high-risk genotypes (**Table 1**). A percentage of samples, although positive for control DNA, were negative for the HPV genotypes included in our detection kit. Regarding age, the CIN3 bearing patients were significantly younger than the other groups. Other known risk factors for cervical cancer did not vary among groups: smoking habits, parity, hormonal contraceptive, number of sexual partners, and age of first intercourse (data not shown).

**Table 1:** Patients’ cohort description.

Sample size	53
CIN3	26
SCC	20
AdC	7
Healthy donors	11
Age	Average (st dev)
CIN3	39 (29–67)
SCC	54 (25–78)
AdC	50 (35–88)
Healthy donors	51 (32–76)
FIGO classification	% (absolute numbers)
I	33.3% (9)
II	11.1% (3)
III	11.1% (3)
IV	11.1% (3)
HPV genotype	% (absolute number)
16	30.1% (16)
18	11.3% (6)
High-risk (16 or 18)	20.7% (11)
Low-risk	0
Multiple infection	5.6% (3)^a^
Negative	13% (7)
No genotype identification	18.8% (10)
Enrollment	Local
ICESP	26
Hospital Universitário	38^b^

^a^Multiple infection with high-risk genotypes; ^b^includes controls subjects.

**Figure 1:**
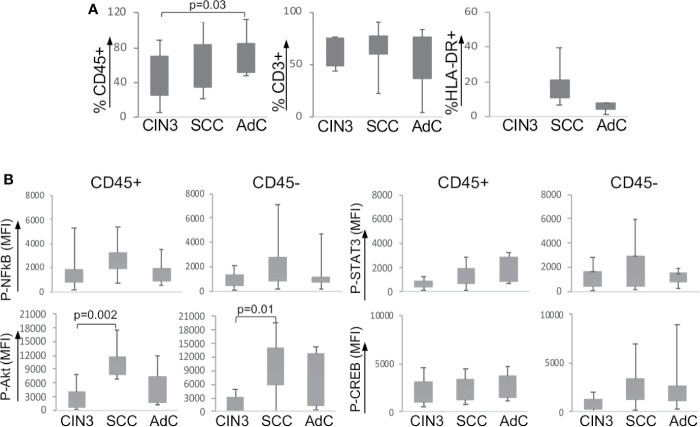
Expression of signaling proteins in cervical biopsies. **(A)** Frequency of infiltrating leukocytes in cervical biopsies. Cells suspensions labeled with anti-CD45, anti-CD3, and anti-HLA-DR were analyzed by flow cytometry, where we acquired at least 10,000 events per sample. Experimental groups were CIN3 – biopsies from patients with high-grade lesion, squamous cell carcinomas (SCC), and adenocarcinomas (AdC) – biopsies from patients with squamous cell carcinoma and adenocarcinoma, respectively. **(B)** Tumor cell suspensions labeled with anti-CD45 to identify CD45- and CD45+ cells were fixed, permeabilized, and intracellularly labeled with antibodies against the indicated phosphorylated proteins. Data is represented as the median of fluorescence intensity (MFI) in boxplots. The indicated values are the signal obtained for each protein minus the MFI values of the unstained controls. Significant differences are indicated in the figure. Experimental groups contained: CIN3 - 7, SCC 13, and AdC – 5 samples.

Our first step was to evaluate the frequency of leucocyte infiltration in the tumor biopsies (**Supplementary Figure 2A** shows the gate strategy analysis for immune cell populations). As we have already shown in a previous study (20), our current data also showed that cervical cancer samples displayed higher leukocyte infiltration (CD45+) than CIN3 samples (**Figure 1A**). Within the leukocyte population (CD45+), we did not observe significant changes in the frequency of T lymphocytes (CD3+) or antigen-presenting cells (CD3-HLA-DR+). As it was not possible to acquire normal cervical tissue, we performed stratified analyses only between CIN3, SCC, and AdC biopsies. Next, we investigated the expression and phosphorylation of the intracellular signaling proteins p65 NFκB, Akt, STAT3, and CREB among the samples (**Figure 1B**). Except for the higher phospho-Akt expression observed in SCC compared to CIN3, there were no other significant alterations in the phosphorylated status of these proteins in the tumor microenvironment, related to lesion grade. All samples displayed similar levels of protein phosphorylation, both in the leukocyte (CD45+) compartment as in other tumor cells (CD45-). Total protein expression also displayed similar levels between CIN3 and cancer samples, with exception of p65 NFκB, which expression was lower in AdC, and Akt, with a similar pattern as observed with the phosphorylated protein (**Supplementary Figure 2B**).

**Figure 2:**
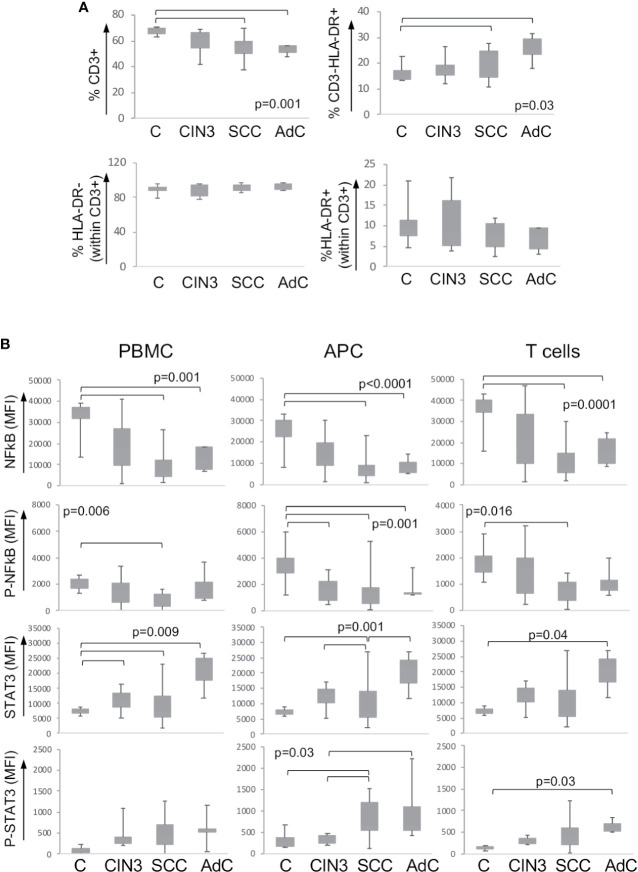
Expression of signaling proteins in circulating leukocytes. **(A)** Frequency of leukocyte populations in the peripheral blood. Peripheral blood mononuclear cells were isolated by density gradient, and cells were labeled with anti-CD3 and anti-HLA. At least 10,000 events were acquired per sample using a FACSCanto. Populations were defined as shown in **Supplementary Figure 2C**. Data are represented as the percentage of the measured population in relation to the total population in the upper plots, and as the percentage within the CD3+ T lymphocyte population in the lower plots. Experimental groups were: C – healthy donor controls, CIN3 – patients with high-grade lesions, squamous cell carcinomas (SCC), and adenocarcinomas (AdC) – patients with squamous cell carcinoma and adenocarcinomas. Significant differences among experimental groups are indicated in the graphs. **(B)** STAT3 and p65-NFkB proteins expression in circulating leukocyte populations according to lesion grade. Peripheral blood mononuclear cells (PBMCs) were labeled with anti-CD3 and anti-HLA-DR, then fixed and permeabilized and intracellularly stained with antibodies against the indicated proteins. Cells were analyzed in a FACSCanto, where, at least 10,000 events were acquired per sample. Data is represented as boxplots using the median fluorescence intensity (MFI) value of each sample minus the background signal obtained with a sample incubated only with the secondary antibody. Experimental groups are: C – healthy donors controls, CIN3 – patients with high-grade lesions, SCC and AdC – patients with squamous cell carcinoma and adenocarcinomas. Significant differences among experimental groups are indicated by bars and corresponding p values. Sample sizes for STAT3 and NFκB: CIN3 n=11, SCC=15, AdC=6. In all cases, we had 11 healthy donor controls.

As mentioned before, we expected to find systemic alterations triggered by cervical lesions. To investigate that, we also analyzed the expression and phosphorylation status of the signaling proteins described above, in the peripheral blood mononuclear cells from our patients’ cohort together with a control group of clinically healthy donors. **Supplementary Figure 2C** shows one example of the gating strategy for the identification of mononuclear cell populations. First, we found a significant decrease in the T cell population (CD3+) and increase in antigen-presenting cells (CD3-HLA-DR+) frequency in cancer patients compared to clinically healthy controls (C) (**Figure 2A**). In spite of that, within the T cell population, there was no difference between activated (CD3+HLA-DR+) and resting T cells (CD3+HLA-DR- cells).

Next, we analyzed the protein expression and the phosphorylation status within the total peripheral blood mononuclear cells (PBMC), antigen-presenting cells (APC – correspondent to CD3-HLA-DR+ cells), and T cells (total CD3+ population). We observed a clear pattern of downregulation of p65 NFκB and upregulation of STAT3 total protein expression and phosphorylation among the high-grade cancer patients compared to healthy donors (**Figure 2B**). Besides that, we found a significant increase in the expression of total Akt in the T cell population in cancer patients and phosphorylated-CREB in patients with CIN3 compared to healthy donors (**Supplementary Figure 3**).

In both peripheral blood and biopsy samples, there was a reasonable spread in the protein expression data. This was not surprising, since these are human samples, and biopsies are from an anatomical site subjected to hormonal changes and exposed to environmental factors. Therefore, we may have missed significance in some situations that might be achieved had we been able to collect more samples. For example, phosphorylated-STAT3 expression seemed to be higher in CD45+ cells in cancer samples than in CIN3 biopsies (**Figure 1B**), which would correlate with the pattern observed in blood samples (**Figure 2B**). Another similarity was the downregulation of p65-NFκB in cancer patients in both peripheral blood samples and biopsies (**Figure 2B**). Therefore, there was a possibility that protein expression in the cancer leukocyte compartment and cells in the peripheral blood could display similarities that we could not see by evaluating expression dependent on lesion grade.

**Figure 3:**
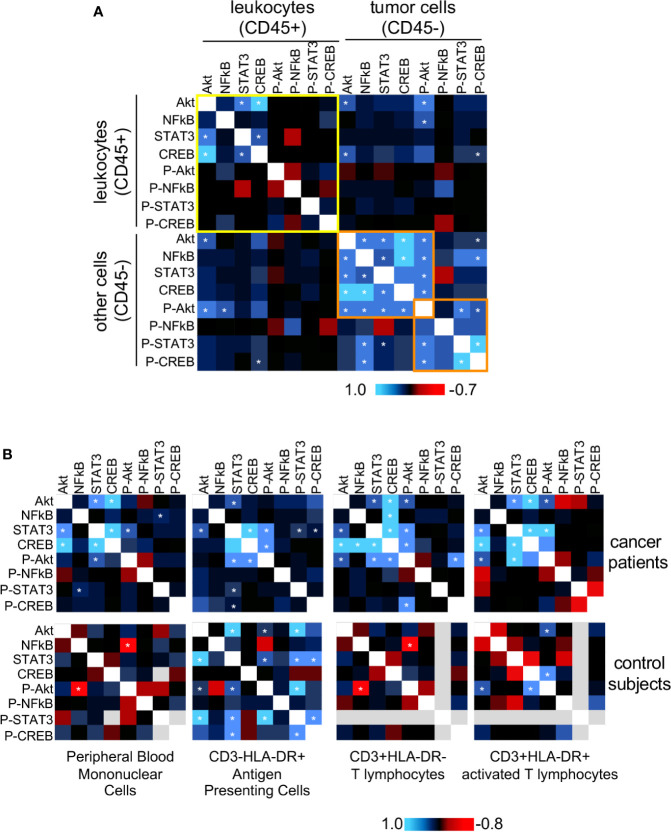
Protein expression and phosphorylation status correlation between tumor and blood leukocytes. For both tumor cells and circulating leukocytes, we used Pearson correlation to compare the expression data (MFI value) of all evaluated proteins, pairwise (data showed in **Figures 1** and **2**). We pooled together data from squamous cell carcinomas (SCC), and adenocarcinomas (AdC) patients for statistical power. For these analyses, we used data from patients with the complete panel of antibody labeling, and therefore had to exclude from the analysis patients with incomplete data. A total of 18 cancer patients were analyzed regarding their biopsy and peripheral blood data. **(A)** Biopsy data, where we highlighted in yellow the correlations within the infiltrating leukocyte population (CD45+), and in orange, a cluster including most of the statistically significant correlation values within the non-leukocyte population, mainly tumor cells (CD45-). **(B)** Peripheral blood data. Top panels are related to cancer patients’ samples and bottom panels to clinically healthy controls data (n=10). In healthy donor samples, some proteins could not be labeled therefore had to be excluded from the analysis (gray areas). Specific leukocyte populations within the peripheral blood mononuclear cells are specified in the figure. * indicates significant correlations. P- indicates phosphorylated proteins.

The proteins we chose to study not only are involved in biological processes important for cancer progression, but they may display interactions among themselves and with other factors within cells. Trying to access the potential interactions among these proteins, we decided to use the protein expression data we already had and calculate if each protein expression was correlated with all others. We used the MFI data to perform Pearson correlations and plotted the values as a heatmap. In **Figure 3**, we show the correlations found from protein expression data from biopsies populations in A, and peripheral blood populations in B. In **Figure 3B** we also showed data from peripheral blood populations from clinically healthy donors. If our premise that biochemical signals generated in the tumor microenvironment could trigger signaling pathways locally and systemically, we should be able to observe similar protein expression correlations in leukocytes within the tumor microenvironment and circulating ones.

In cancer biopsies (**Figure 3A**), it was clear that leukocytes (CD45+) and cancer cells (CD45-) displayed different protein expression correlation patterns. In CD45- cells there were significant positive correlations among the expression of all total proteins, and there was a positive correlation among phosphorylated-Akt, phosphorylated-CREB, phosphorylated-STAT3 expression (highlighted in orange). There was also a positive correlation between NFκB and phospho-CREB expression. Among the leukocytes (CD45+) (highlighted in yellow), however, we only observed significant positive correlations among the expression of total Akt, STAT3, and CREB. These results indicated that, although, in the same microenvironment, different cell populations integrated signals differently, depending on their lineage, as expected.

In peripheral blood samples (**Figure 3B**), as mentioned before, we had the opportunity to compare data from cancer patients and clinically healthy controls. First, regarding the expression correlation patterns among cell populations, either in cancer patients or controls. We could observe that each analyzed population (antigen-presenting cells, resting or activated T cells), had a distinct expression correlation pattern. For example, in all cell populations from cancer patients, there was a positive expression correlation between STAT3 and Akt. However, while there was a positive correlation between p65 NFκB and CREB expression in T cells, the same was not true in antigen-presenting cells or activated T lymphocytes. Similarly, in controls, we could observe a significant negative correlation between phosphor-Akt and NFκB, but not in the other populations, just to mention one example. The PBMCs pattern was mainly similar to the pattern observed in T lymphocytes (CD3+HLA-DR-), in both cancer patients and control subjects blood samples. This was to be expected since among the populations analyzed, T lymphocytes were the most abundant within PBMCs.

Interestingly, as observed in the CD45+ population in cancer biopsies, in PBMCs from cancer patients, there was a positive correlation between Akt and STAT3 and CREB expression. These correlations were not found in control subjects’ PMBCs, where we found, instead, a negative correlation between p65 NFκB and phospho-Akt.

The most interesting point to take from **Figure 3**, however, came from the observation that expression patterns in circulating leukocytes from cancer patients were more similar to the pattern in cancer infiltrating leukocytes, than to the pattern observed in peripheral blood cells from clinically healthy donors (**Figure 3B** upper panels – cancer patients, lower panels – controls). These data clearly showed that cervical cancer triggers alterations systemic signaling, indicating that cells recruited to the tumor microenvironment already have a biased phenotype.

### STAT3 Promotes Tumor Growth Through Immune Response Suppression, in A Mechanism Partially Dependent on NFκB Downregulation

**Figure 4:**
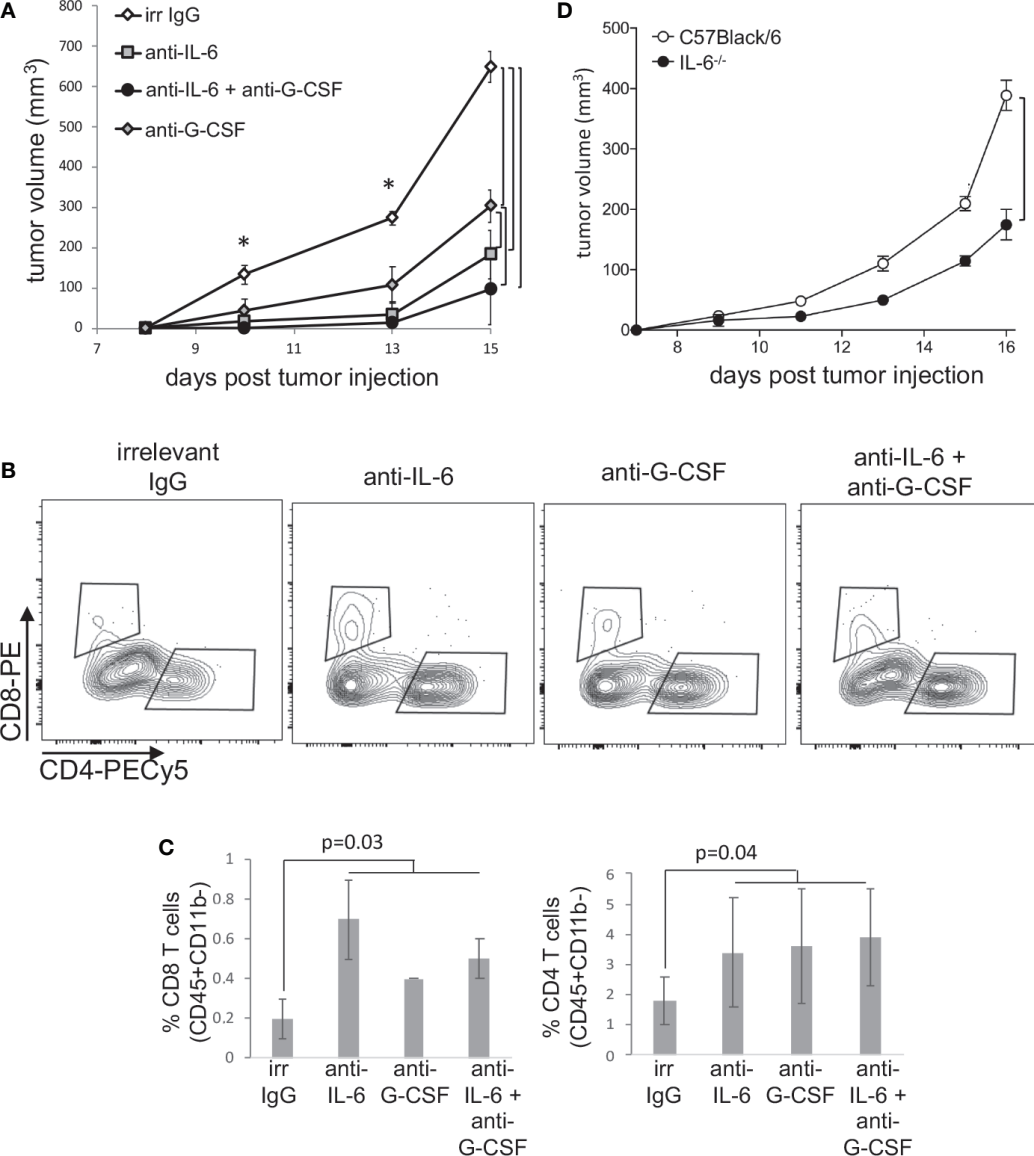
Neutralization of IL-6 and G-CSF inhibits tumor growth, through changes in immune responses. **(A)** Tumor-bearing C57Black/6 mice were treated with 50 µg of anti-IL6 or IL-G-CSF or combination of both every other day. Controls were tumor-bearing mice treated with an equivalent concentration of irrelevant IgG (irr IgG). Tumor growth kinetics is represented by tumor volumes measured with a pachymeter (volume = D*d^2^/2, where D is the largest diameter and d is the smallest diameter). **(B, C)** Tumor cell suspensions, from mice described in **(A)**, were labeled with antibodies to determine T cells infiltration by flow cytometry. After debris and doublets exclusion, we gated on CD45+ CD11b- cells for the identification of CD4+ and CD8+ T lymphocytes. **(B)** shows a representative experiment and **(C)** the quantification of data from two experiments with a total of eight mice. **(D)** TC-1 cells were injected in IL-6^-/-^ mice and control C57Black/6 mice. Tumor measurements were performed as described above. Significant differences indicated by bars or corresponding p values, in **(A)** * also mark significant differences between control group and the others at specific time points. Even if not specified, p<0.05. Tumor growth kinetics were compared by the Mann-Whitney U test and the T cell infiltration by ANOVA.

The results described above clearly showed that cervical cancer displayed systemic effects on cancer patients’ immune system. The robust effect we observed in the upregulation of STAT3 expression dependent on lesion grade could be related to inflammatory cytokine signaling. Others and we have shown that HPV transformed cells, both human and murine; secrete IL-6 and G-CSF (4, 7, 19–21), both inducers of STAT3 activation (29). Our patients’ data together with experimental evidence that chronic activation of STAT3 promotes tolerance toward cervical cancer antigens (5, 8) led us to investigate if IL-6 and G-CSF could have a role in tumor growth and modulation of tumor systemic effects. We used the HPV16 associated murine tumor model, TC-1 (24), to test this hypothesis. We treated TC-1 tumor-bearing mice with neutralizing anti-G-CSF and/or anti-IL-6 and observed a significant decrease in tumor growth compared to the control group treated with irrelevant antibody (**Figure 4A**). The tumor growth inhibition was even more effective when the animals were injected with both antibodies simultaneously (**Figure 4A**). Cytokine neutralization also promoted a significant increase in the frequency of tumor-infiltrating T lymphocytes: at least 2-fold for both CD4 and CD8 T cells (**Figures 4B, C**). We did not observe significant differences in the frequency of tumor-infiltrating myeloid cells or total leukocyte infiltration (data not shown). Systemically, neutralization of IL-6 and G-CSF led to a decrease in the frequency of CD11b+Ly6C+Ly6G+ myeloid cells in the spleen (**Supplementary Figures 4A, B**) and also reverted the tumor triggered inhibition of NFκB in antigen-presenting cells and T lymphocytes (**Supplementary Figure 4C**). Because antibody treatment can promote off-target effects and face distribution resistance inside of the tumor microenvironment to block cytokine signaling, we decided to inject TC-1 tumor cells an IL-6 deficient mice, where was observed a significant reduction in the TC-1 tumor growth compared to wild type mice growth curve (**Figure 4D**), confirming IL-6 role as a pro-tumoral signal.

**Figure 5:**
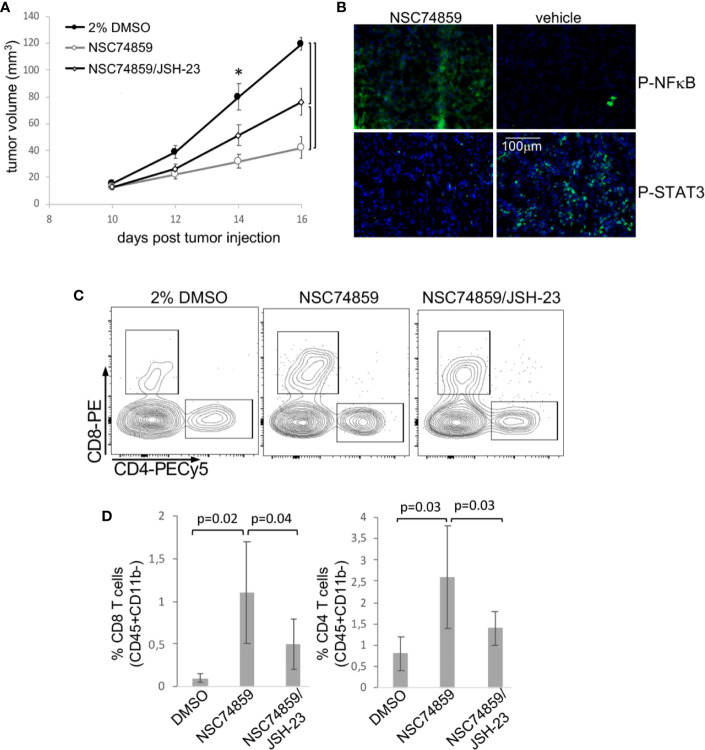
STAT3 inhibition inhibits tumor growth and promotes anti-tumor T cell responses. Tumor-bearing C57Black/6 mice were treated with 5 mg/Kg NSC74859 alone or combined with 3 mg/Kg JSH-23 every day from day 9 post tumor cell inoculation until day 16. Controls were treated with 2% DMSO in phosphate-buffered saline (PBS), which was de dilution solution for the inhibitors. **(A)** Tumors were measured with a pachymeter to determine tumor volume (volume = D*d^2^/2, where D is the largest diameter and d is the smallest diameter). **(B)** Expression of phosphorylated STAT3 and p65 NFκB proteins in tumors detected by immunofluorescence. Cryosections from fresh tumors from animals treated as described above were labeled with antibodies against the indicated targets (fluorescence obtained from secondary Alexa 488 anti-rabbit antibody) and counterstained with DAPI. **(C, D)** Tumor single-cell suspensions were used to determine the frequency of tumor-infiltrating T lymphocytes by flow cytometry. After debris and doublets exclusion, we gated on CD45+CD11b- cells to identify the CD4+ and CD8 T lymphocytes. **(C)** Shows a representative experiment. **(D)** Shows the quantification of data from four experiments with a total of 12 mice. Significant differences indicated by bars or corresponding p values in **(A)** * also mark significant differences between control group and the others at specific time points. Even if not specified, p<0.05. Tumor growth kinetics were compared by the Mann-Whitney U test, and the T cell infiltration by ANOVA.

Both IL-6 and G-CSF, through their specific receptors can activate more than one signaling pathway. For example, both can ctivate the PI3K/Akt pathway that, as previously shown, was activated in both tumor microenvironment and circulating leukocytes. Therefore, to test if signaling through STAT3 was important for tumor growth and systemic effects, we used the STAT3 inhibitor, NSC74859 (30) to treat tumor-bearing mice. Mice were injected with 5mg/Kg NSC74859 daily for 7 to 9 days. STAT3 inhibitor treatment significantly decreased tumor growth (**Figure 5A**). We also observed that the treatment inhibited the expression of phosphorylated-STAT3 and increased the expression of phosphorylated-p65-NFkB in comparison to control tumors (**Figure 5B**). Moreover, treatment significantly increased T lymphocyte tumor infiltration, both CD4 and CD8 T cells (**Figures 5C, D**). Again, there were no significant differences in the myeloid populations infiltrating tumors from the different experimental groups (data not shown).

In patients, we observed that cancer progression was associated with both increase in STAT3 expression and decrease in NFκB expression in peripheral blood cells. Added to that, in our experimental tumor model, the inhibition of STAT3 increased p65-NFκB expression in TC-1 tumors (**Figure 5B**), which could be a direct or indirect mechanism. That led us to test whether STAT3 mediated tumor growth and evasion mechanisms could involve p65-NFκB modulation. We treated tumor-bearing mice with a combination of STAT3 inhibitor and NFκB inhibitor, JSH-23. Treatment with the NFκB inhibitor partially reversed the effects observed with STAT3 inhibition regarding both tumor growth and T lymphocyte tumor infiltration (**Figures 5A, C, D**). This result indicated that part of the STAT3 pro-tumoral effect involves the inactivation of NFκB. We cannot tell at this point if this is a direct or indirect effect.

As in tumors, treatment with STAT3 inhibitor also promoted downregulation of phosphorylated STAT3 expression systemically, while causing phosphorylated p65-NFkB expression to increase to levels similar to those observed in spleens from naïve mice (**Supplementary Figure 5A**). Moreover, TC-1 tumors caused CD11b+Ly6C+ and CD11b+Ly6C+Ly6G+ myeloid cell accumulation in the spleen. But this effect was completely abrogated by treatment with the STAT3 inhibitor (**Supplementary Figure 5B**). Treatment did not affect on the frequency of spleen T lymphocytes (CD3+) (**Supplementary Figure 5B**).

**Figure 6:**
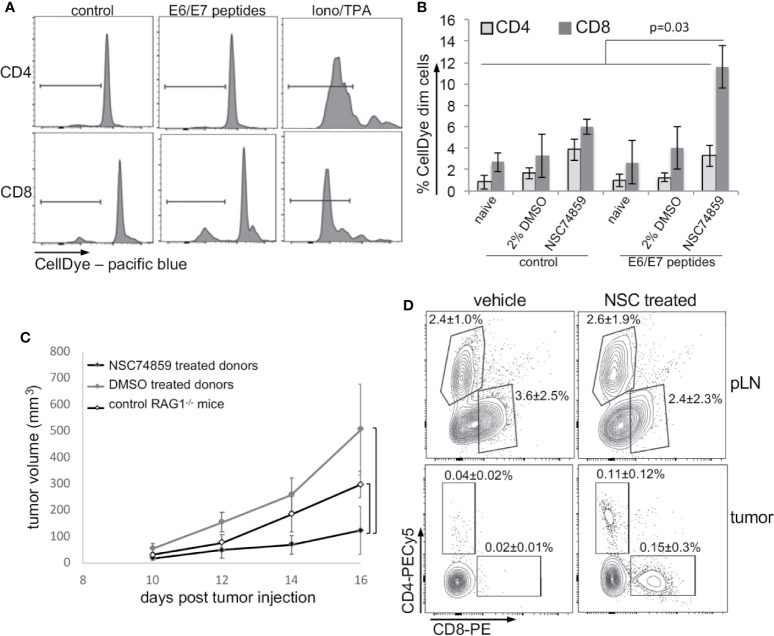
STAT3 inhibition increases antigen-specific anti-tumor responses. **(A, B)** T lymphocyte proliferation assay. Lymph node cell suspensions (stained with violet proliferation dye) were treated with 5 µg/ml HPV16 E6 and E7 peptides or 1µg/ml ionomycin and 10 ng/ml TPA for 4 days and then labeled with anti-CD4 and anti-CD8 and analyzed by flow cytometry. **(A)** Cell proliferation representative experiment. **(B)** Quantification of data from a total of five mice per experimental group. Data is presented as the percentage of cells that diluted the CellDye and compared by ANOVA. **(C)** Lymph node single-cell suspensions were transferred from 2% DMSO or NSC74859 treated tumor-bearing mice donor to tumor-bearing RAG1^-/-^ recipient mice. A group of recipients was not reconstituted to be used as controls. Tumors were measured for 5 days and, by the end of this period peripheral lymph nodes and tumors were harvested from RAG1^-/-^ recipient mice. **(D)** Tumors were processed to determine the frequency of tumor-infiltrating T lymphocytes. The average frequencies of infiltrating CD4 or CD8 T lymphocytes indicated in the graphs refer to the total number of tumor cells. As controls, the frequency of CD8 and CD4 T lymphocytes in the peripheral lymph nodes of transplanted mice is shown. A total of six reconstituted mice per group were analyzed.

Our results up to this point indicated that STAT3 inhibition could impair tumor growth, increase tumor T lymphocyte infiltration, and decrease tumor systemic effects. An increase in tumor T lymphocyte infiltration could reflect activation of antigen specific anti-tumor immune responses. To test if this was the case, we stimulated cells from peripheral lymph nodes from tumor-bearing mice treated with NSC74859, tumor-bearing mice without any treatment, and from naïve mice with peptides correspondent to E6 and E7 MHC-I epitopes. T lymphocyte activation was measured by dilution of a cell proliferation dye, as exemplified in **Figure 6A** and quantified in **Figure 6B**. As we can observe, inhibition of STAT3 promoted antigen-specific CD8 T lymphocyte proliferation (**Figure 6B**) indicating that treatment enabled the activation of cytotoxic immune responses. To confirm this hypothesis, we transferred lymph node cell suspensions from tumor-bearing C57Black/5 mice donors to tumor-bearing RAG1-/- recipient mice. Recipient mice that received cells from donors treated with STAT3 inhibitor displayed impaired tumor growth compared to both mice that receive lymphocytes from control tumor-bearing mice or non-reconstituted RAG1-/- mice (**Figure 6C**). Importantly, transferred cells from treated and control tumor-bearing donors had the same potential to engraft mice recipient (**Figure 6D** - upper dot plots). However, we found more T lymphocytes in tumors from mice that were transplanted with cells from STAT3 inhibitor-treated donors (**Figure 6C** - bottom dot plots). These results indicated that STAT3 inhibition increased anti-tumor immune responses, possibly allowing the generation of memory T lymphocytes.

## Discussion

Tumor cells are dependent on the microenvironment for survival, growth, invasion, and even resistance to drugs (31). Data in the literature indicate that solid tumors display systemic effects, including alteration in leukocyte circulating populations. There are two important questions that this observation raises: 1) if leukocytes recruited to the tumor microenvironment are already biased toward a pro-tumoral phenotype due to systemic tumor effects; 2) if these altered cells have any role in modulating immune responses systemically.

Dissecting the roles of individual signaling pathways in the tumor ecosystems is complex because it is difficult to distinguish the source of the reciprocal paracrine and endocrinal signals as a consequence of the interactions between populations of neoplastic cells and tumor-associated inflammatory cells. Moreover, it has been described that HPV transformed cells display activated p65-NFκB, STAT3, and Akt (8, 32–35). Indeed, we found positive expression of all proteins studied in CIN3 and cancer biopsies, in both the CD45+ and CD45- cell compartments. Besides, when we compared the expression levels (MFI values) between each protein and all others by Pearson correlation, we found a correlation pattern in CD45+ cells different than the correlation pattern in CD45- cells. This was to be expected since tumor cells and leukocytes are completely different cells and would respond to the environment in different manners. Moreover, tumor cells express HPV oncoproteins that trigger several signals in tumor cells, as mentioned before. In HPV associated tumor cells, STAT3 expression may be indirectly activated by NFκB, since IL-6 is a transcription target of the later and known activator of the STAT3 pathway. This seems to be the case in cervical cancer cells according to Morgan and Macdonald’s data, which could explain the positive correlation between the expression of these two proteins in tumor cells (CD45-). Of notice, HPV oncoproteins E6 and E7 can activate NFκB and Akt, which corroborate our data related to cancer cells (7, 33).

The blood neutrophil to lymphocyte ratio (NLR) in cancer patients has been used as a prognostic marker for many types of cancer, including cervical cancer (36–39). What our data points out is that not only circulating cell populations’ frequency was altered in cancer patients, but their phenotype is also changed. Data in experimental models have been showing this for a long time. Observation of accumulation of myeloid-derived suppressor cells in the spleen of tumor-bearing mice, for instance, reflexes our observations (8, 9, 40). The very clear and significative increase in STAT3 and decrease in NFκB expression in circulating leukocytes not only can evidence the systemic effects of cervical cancer, as actually may have an important biological significance.

**Figure 7:**
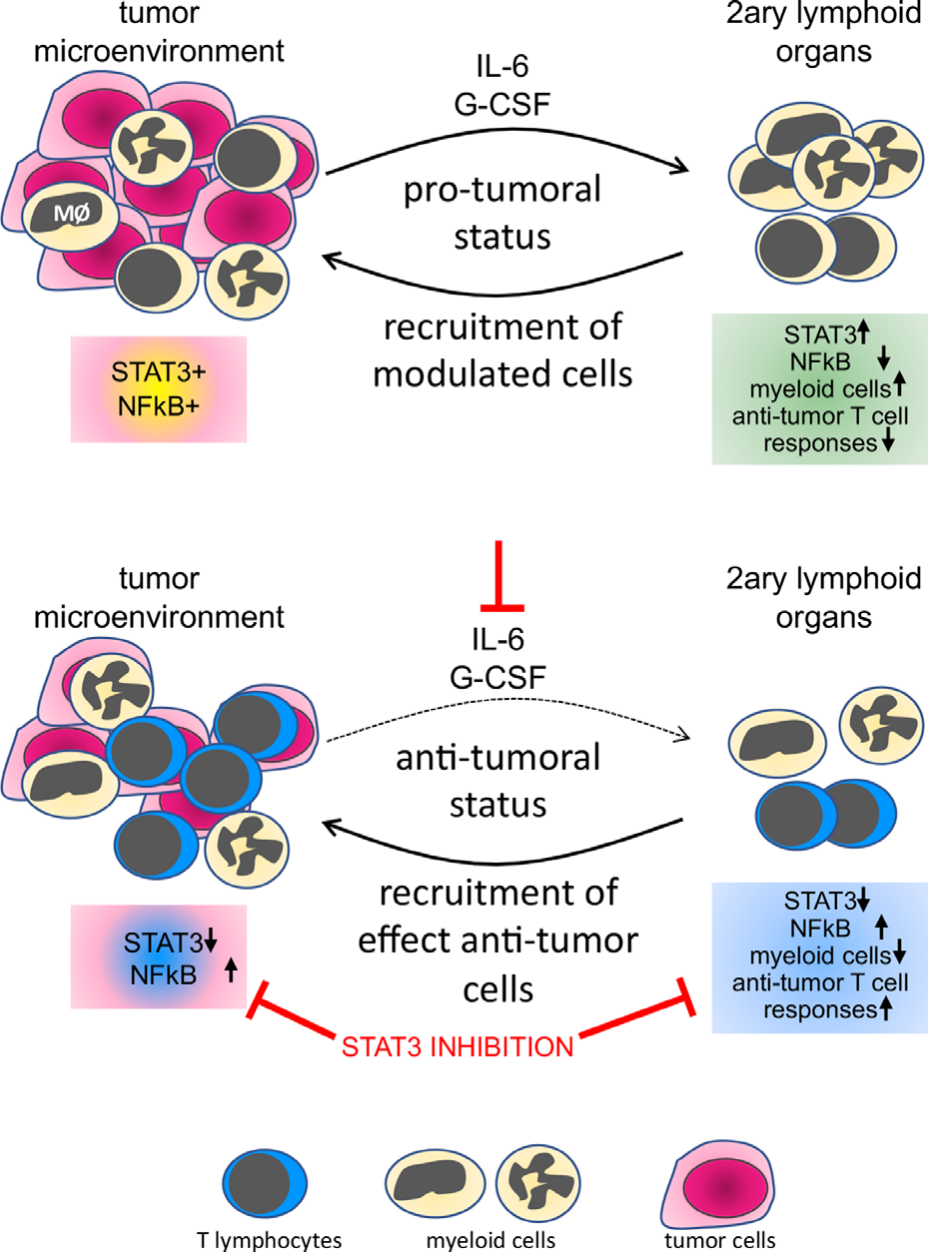
Representation of the tumor systemic effects on immune cells and the result of STAT3 inhibition. In the top panel, we show that both leukocytes and tumor cells express STAT3 and p65NFκB, both phosphorylated, indicating activation. Factors secreted by cells in the tumor microenvironment, as IL-6 and G-CSF, signal systemically controlling not only leukocyte populations frequency but also phenotype, through an increase in STAT3 expression and decrease in p65 NFκB expression in secondary lymphoid organs or peripheral blood. In the lower panel, neutralization of IL-6 and G-CSF or inhibition of STAT3 impairs tumor growth, reduces the frequency of myeloid cells in secondary lymphoid organs, and increases anti-tumor T cells responses.

HPV transformed cells secrete IL-6 and G-CSF, as shown by different research groups (5, 9, 20). Macrophages in the tumor microenvironment also secrete IL-10. All these cytokines activate the STAT3 pathway (41). Some researchers consider STAT3 an oncogene, with a role in cellular proliferation, survival, and epithelial/mesenchymal transition (42). STAT3 activity is also part of the molecular mechanism leading tolerance toward tumor antigens (43). Systemically, it can promote tolerance toward tumor antigens through the increase in the frequency of myeloid-derived suppressor cells (8). The ability of STAT3 signaling networks to integrate and promote regulatory roles in both, tumor cells and immune cells suggests that this pathway must act as a signaling node, responding to multiple inputs and regulating different effector outputs. Within the evaluated leukocyte populations, we found that the protein expression correlation pattern observed in peripheral blood cells, mainly T lymphocytes, was more similar to the pattern observed in the CD45+ population in cancers than in leukocytes from clinically healthy donors. These results are proof of the modulatory effect that tumors can have on circulating leukocytes, indicating that cells recruited to tumors are already biased by the tumor microenvironment (**Figure 7**).

Our data showing the opposite expression of STAT3 and p65-NFκB according to lesion grade in peripheral blood leukocytes led us to speculate whether these signaling pathways could be part of the immunomodulatory effects of cervical cancer. Of notice, we did not observe a negative correlation between these proteins expression in the analysis displayed in **Figure 3**. However, it is important to differentiate, direct protein expression correlation in cancer samples (**Figure 3**), and expression variation according to lesion grade (**Figure 2**). In our mouse tumor model, STAT3 inhibition led to an increase in phoshorylated-p65-NFκB expression, both in the tumor as in the spleen. This could be due to the loss of direct STAT3 interference on NFκB activation. However, these protein expression patterns in both the tumor and spleen were too different for this hypothesis to be the case. While phosphorylated-STAT3 expression per cell was high, it had a discrete expression pattern relatively well distributed throughout the tumor and spleen tissue (**Figure 5** and **Supplementary Figure 4A**). Phospho-p65 NFκB expression, on the other hand, displayed more diffuse expression in heterogeneous tumor and spleen areas. This indicated that switch in protein expression triggered by treatment was occurring in different cells. Therefore, either STAT3 could generate paracrine signals capable of inhibiting NFκB activation; or tumors could inhibit NFκB, through other mechanisms that do not directly involve STAT3, but that were reduced as treatment with STAT3 inhibition reduced tumor growth. For example, we have shown that lactate, a metabolite secreted by cervical cancer cells downregulated phosphorylated-p65-NFkB expression (35). We did not investigate the expression of p50-NFκB. It is possible that in cells from tumor-bearing hosts we might find p50/p50 homodimers, which may suppress inflammatory and immune responses (44). In the past, we showed that IL-10, secreted by tumor-associated macrophages, could reduce phosphorylated-p65-NFκB expression in splenocytes of tumor-bearing mice as part of the tumor evasion mechanism (19). IL-10 also signals through STAT3 to inhibit inflammatory and immune responses, corroborating the data presented in this manuscript (45).

Pharmacologic and antibody-based inhibitors that target signaling proteins in tumors have had a significant impact on cancer treatments. The use of STAT3 inhibitors for cancer treatment has been discussed in the literature for some time. Several ongoing clinical trials are using different approaches for STAT3 inhibition in cancer patients. There are, however, indications that the use of STAT3 inhibitors could hamper immune responses, which would be expected, mainly for Th17 responses. In the specific case of cervical cancer, there is no consensus regarding the role of CD4 Th17 lymphocytes. Punt and collaborators have shown that a high number of Th17 in cervical cancer improved prognosis, while high IL-17 expression by granulocytes and innate lymphoid cells represented poor prognosis (46). Interestingly, there is also data indicating that Th17 response can cause chronic inflammation and promote cancer progression (47). Moreover, our experimental data shows that inhibition of STAT3 increases anti-tumor immune responses. Therefore, we suggest that patients with cervical cancer, or even other HPV associated cancers, may benefit from treatment with STAT3 inhibitors, mainly if associated with other treatments, possibly chemo and radiotherapy, but even better combined with immunotherapies that could trigger Th1 immune responses (**Figure 7**).

## Data Availability Statement

The raw data supporting the conclusions of this article will be made available by the authors, without undue reservation.

## Ethics Statements

The studies involving human participants were reviewed and approved by the Ethics Committee in Research, the Ethics Committee of Hospital Universitário and Instituto do Câncer do Estado de São Paulo, process 02083912.6.0000.5467 (National Health Council). The patients/participants provided their written informed consent to participate in this study. The animal study was reviewed and approved by the Ethics Committee in Animal Use at the Instituto de Ciências Biomédicas, Universidade de São Paulo, process 5/2015-E. No potentially identifiable human images or data are presented in this study.

## Author Contributions

RR, GR, IS-J, KA, SS, MC, CS, MB, and GM were responsible for most experimental work. MU, JC, KY, MR, and NL were responsible for patients’ samples harvesting. PM and EB were the responsible personnel for the Gynecology services. LV reviewed the HPV genotyping data and the manuscript text. RR and AL designed the project. AL was responsible for some of the experiments and writing the manuscript. LV, RR, and IS-J reviewed the manuscript. All authors contributed to the article and approved the submitted version.

## Funding

This work and SS, KA, and RR fellowships were supported by Fundação de Amparo à Pesquisa do Estado de São Paulo under grants 2014/19326-6, 2013/26856-9, 2011/20499-4, 2011/11121-8. MC had an undergraduate fellowship 2016/16149-1. GR had a fellowship from Conselho Nacional de Desenvolvimento e Pesquisa, CS had a Comissão de Aperfeiçoamento de Pessoal Nível Superior fellowship. AL has a Conselho Nacional de Desenvolvimento research fellowship 307841/2018-9. This work was supported by FAPESP grants: 2018/16989-5 and 2014/19326-6.

## Conflict of Interest

The authors declare that the research was conducted in the absence of any commercial or financial relationships that could be construed as a potential conflict of interest.

## Supplementary Material

Supplementary Figure 1 Intracellular antibody labeling optimization. After labeling surface antigens, cells were fixed and permeabilized, and then intracellularly labeled with antibodies against total or phosphorylated (P-) Akt, CREB, STAT3, and p65 NFκB proteins. In this figure, we displayed representative histograms related to signaling protein labeling. As negative control cells were incubated with only the FITC conjugated anti-rabbit secondary antibody, and HeLa cells were used as a positive control.

Supplementary Figure 2
**(A)** Example of flow cytometry analysis for protein expression profile in the biopsies populations. After the exclusion of debris and doublets (first two dot plots), the labeling with anti-CD45 allowed us to identify the tumor cell (CD45-) and the leukocytes (CD45+) populations. Within the leukocytes, it was possible to identify two main subpopulations, the antigen-presenting cells (CD3-HLA-DR+) and the T lymphocytes (CD3+). **(B)** Total protein expression in the biopsies. Tumor cell suspensions labeled with anti-CD45 were fixed, permeabilized and intracellularly labeled with antibodies against the indicated total proteins. Data is represented as the median of fluorescence intensity (MFI) in boxplots. The indicated values are the signal obtained for each protein minus the MFI values of the negative controls. Significant differences are indicated in the figure. Experimental groups contained: CIN3 - 7, SCC 13, and AdC – 5 samples. **(C)** Representation of the gating strategy for flow cytometry for the peripheral blood cells. After exclusion of debris and doublets (first two dot plots), we used labeling with anti-CD3 and anti-HLA-DR to identify T lymphocytes (CD3+), activated T lymphocytes (CD3+HLA-DR+), and antigen-presenting cells (CD3-HLA-DR+).

Supplementary Figure 3 Akt and CREB expression in circulating leukocyte populations according to lesion grade. PBMCs were labeled with anti-CD3 and anti-HLA-DR, then fixed and permeabilized and intracellularly stained with antibodies against the indicated proteins. Cells were analyzed in a FACSCanto, where, at least 10,000 events were acquired per sample. Data is represented as boxplots using the MFI value of each sample minus the background signal obtained with a sample incubated only with the secondary antibody. Experimental groups are: C – healthy donor controls, CIN3 – patients with high-grade lesions, SCC and AdC – patients with squamous cell carcinoma and adenocarcinomas. Bars and corresponding p values indicate significant differences between experimental groups. Sample sizes for Akt: CIN3 n=9, SCC=10, AdC=4; for CREB: CIN3 n=7, SCC=10, AdC=3. The samples sized for the AdC group were considered insufficient for analyzes. In all cases, we had 11 health donors.

Supplementary Figure 4
*Systemic effects of IL-6 and G-CSF neutralization*. Spleens were harvested from the mice described in **Figure 4**. Naïve mice and irrelevant IgG treated tumor-bearing mice, when applicable, were used as controls (naive and irr IgG, respectively). Splenocytes were labeled with antibodies and analyzed by flow cytometry to determine the frequency of leukocytes (A and B) and expression of phosphorylated p65-NFκB, after fixation and permeabilization (C). A. Gating strategy to identify spleen populations. To identify the myeloid populations first, we gated in the CD11b+ population, and within this population, we analyzed the Ly6C and Ly6G subpopulations. B. Frequency of splenocytes in naïve or tumor-bearing mice treated with the indicated antibodies. Average of 9 different animals per group is represented in the histograms. C. Phosphorylated-p65 NFκB expression measured by flow cytometry. Splenocytes labeled with anti-MHC-II (APC) and anti-CD3 (T cells), were fixed, permeabilized, and labeled with antibodies against phosphorylated-p65 NFκB. Histograms represent the median fluorescence intensity (MFI). Values indicated on the right side of histograms are the average and standard deviation of the MFI results from 3 mice. The indicated values are the signal obtained minus the MFI values of the unstained control.

Supplementary Figure 5
*Systemic effects of STAT3 inhibition.* A. Phosphorylated STAT3 and p65 NFκB expression in spleen cryosections from different experimental groups (as described in **Figure 6**): – tumor-bearing mice treated with STAT3 inhibitor (NSC748590), tumor-bearing mice treated with 2%, and naïve – control mice without tumor cell inoculation. The negative control is a spleen section incubated only with Alexa 488 conjugated anti-rabbit antibody. B. Frequency of populations in the spleen of the indicated experimental groups. Splenocytes were labeled with antibodies against the indicated cell surface antigens and analyzed by flow cytometry. The histograms indicated the average of each population frequency in the different experimental groups. * indicate significant differences between the control tumor-bearing mice and naïve mice or mice treated with STAT3 inhibitor.
