# Supplementary figures and images for: Local and Systemic STAT3 and p65 NF-KappaB Expression as Progression Markers and Functional Targets for Patients With Cervical Cancer
Source: Front Oncol. 2020 Nov 19;10:587132. doi: 10.3389/fonc.2020.587132 (PMC7710991; doi:10.3389/fonc.2020.587132)

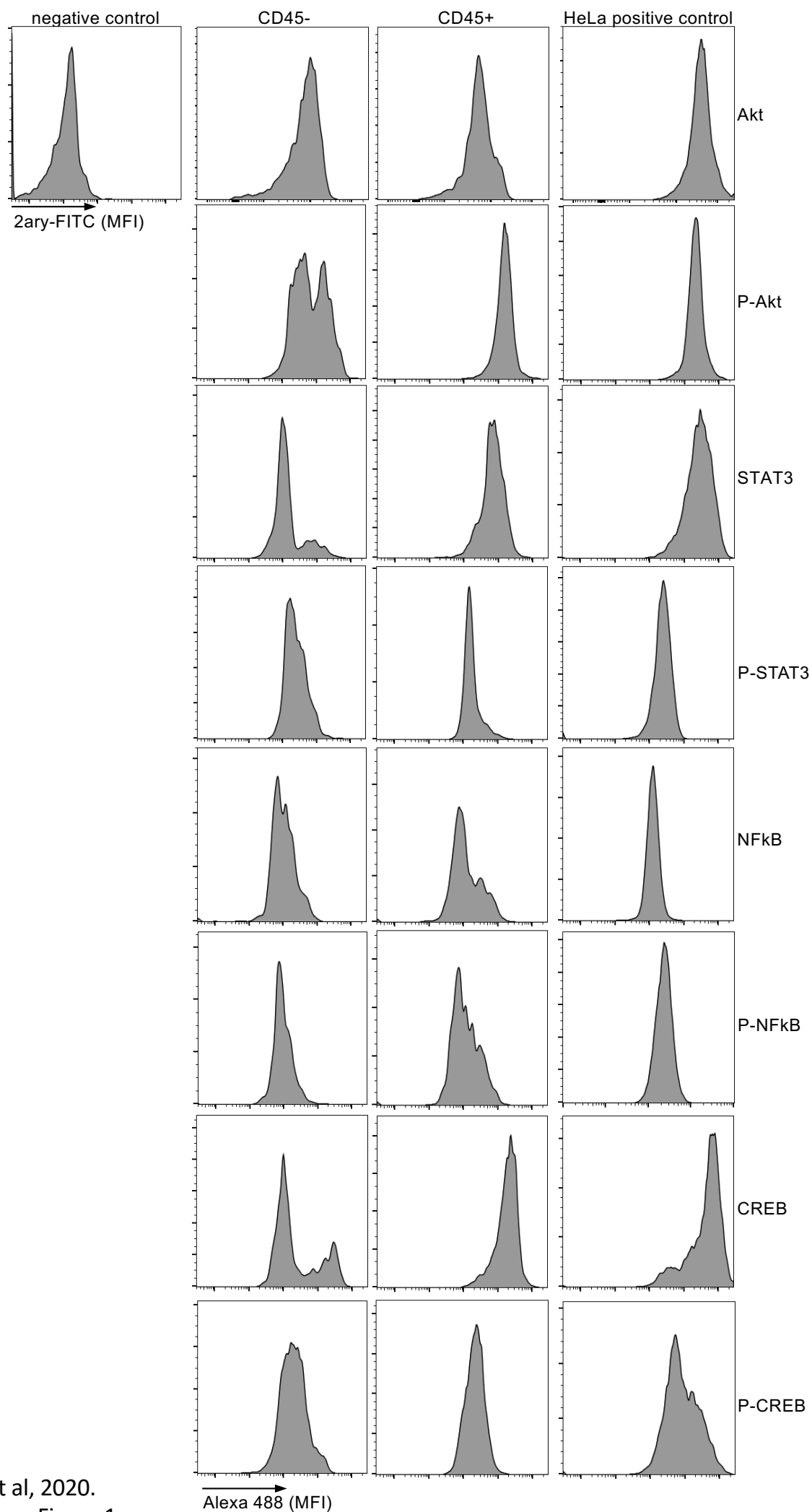

Supplement: Supplementary Figure 1 — Intracellular antibody labeling optimization. After labeling surface antigens, cells were fixed and permeabilized, and then intracellularly labeled with antibodies against total or phosphorylated (P-) Akt, CREB, STAT3, and p65 NFκB proteins. In this figure, we displayed representative histograms related to signaling protein labeling. As negative control cells were incubated with only the FITC conjugated anti-rabbit secondary antibody, and HeLa cells were used as a positive control. [file DataSheet_1.pdf]

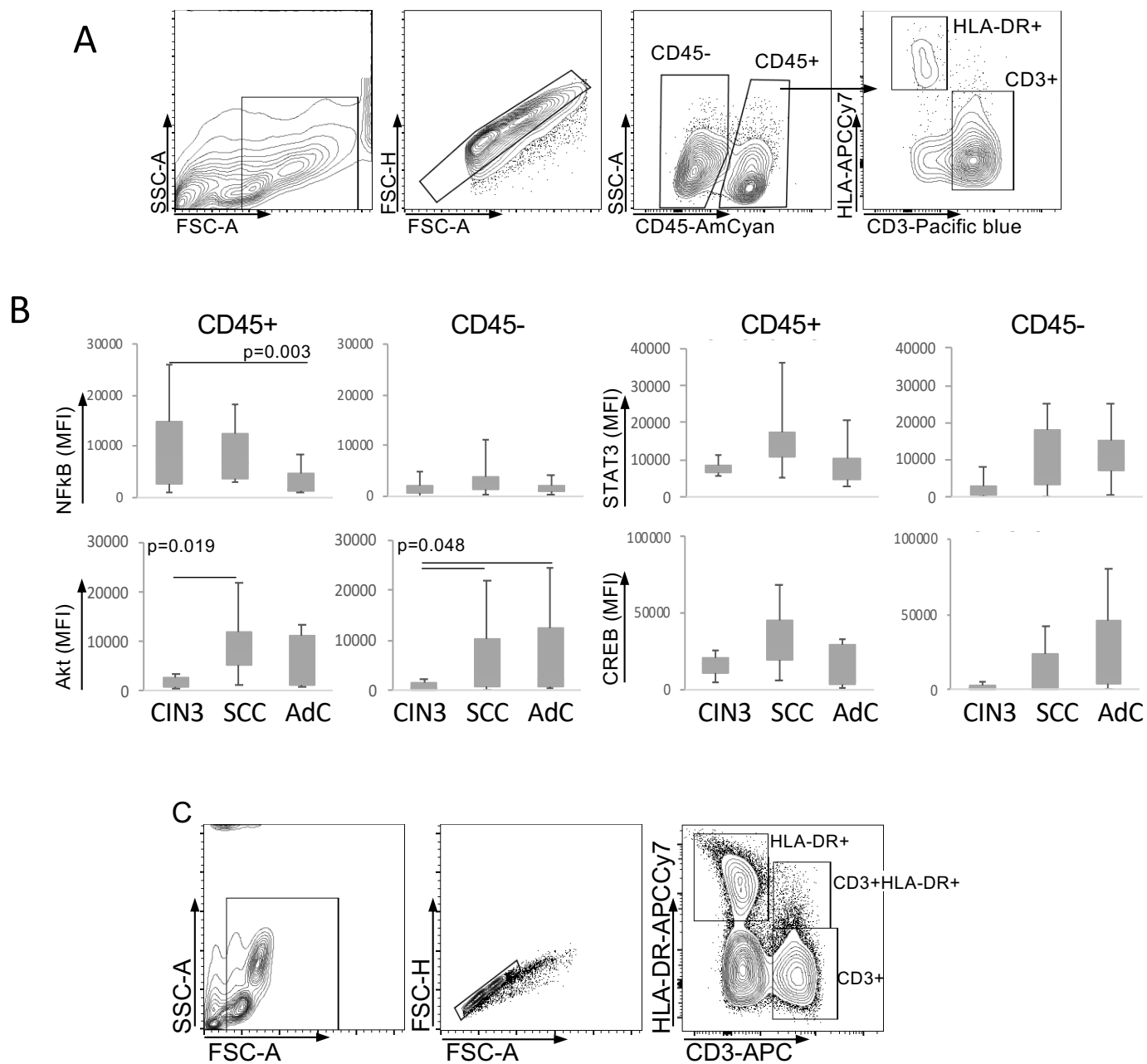

Rossetti et al. 2020  
Supplementary Figure 2

Supplement: Supplementary Figure 2 — (A) Example of flow cytometry analysis for protein expression profile in the biopsies populations. After the exclusion of debris and doublets (first two dot plots), the labeling with anti-CD45 allowed us to identify the tumor cell (CD45-) and the leukocytes (CD45+) populations. Within the leukocytes, it was possible to identify two main subpopulations, the antigen-presenting cells (CD3-HLA-DR+) and the T lymphocytes (CD3+). (B) Total protein expression in the biopsies. Tumor cell suspensions labeled with anti-CD45 were fixed, permeabilized and intracellularly labeled with antibodies against the indicated total proteins. Data is represented as the median of fluorescence intensity (MFI) in boxplots. The indicated values are the signal obtained for each protein minus the MFI values of the negative controls. Significant differences are indicated in the figure. Experimental groups contained: CIN3 - 7, SCC 13, and AdC – 5 samples. (C) Representation of the gating strategy for flow cytometry for the peripheral blood cells. After exclusion of debris and doublets (first two dot plots), we used labeling with anti-CD3 and anti-HLA-DR to identify T lymphocytes (CD3+), activated T lymphocytes (CD3+HLA-DR+), and antigen-presenting cells (CD3-HLA-DR+). [file DataSheet_2.pdf]

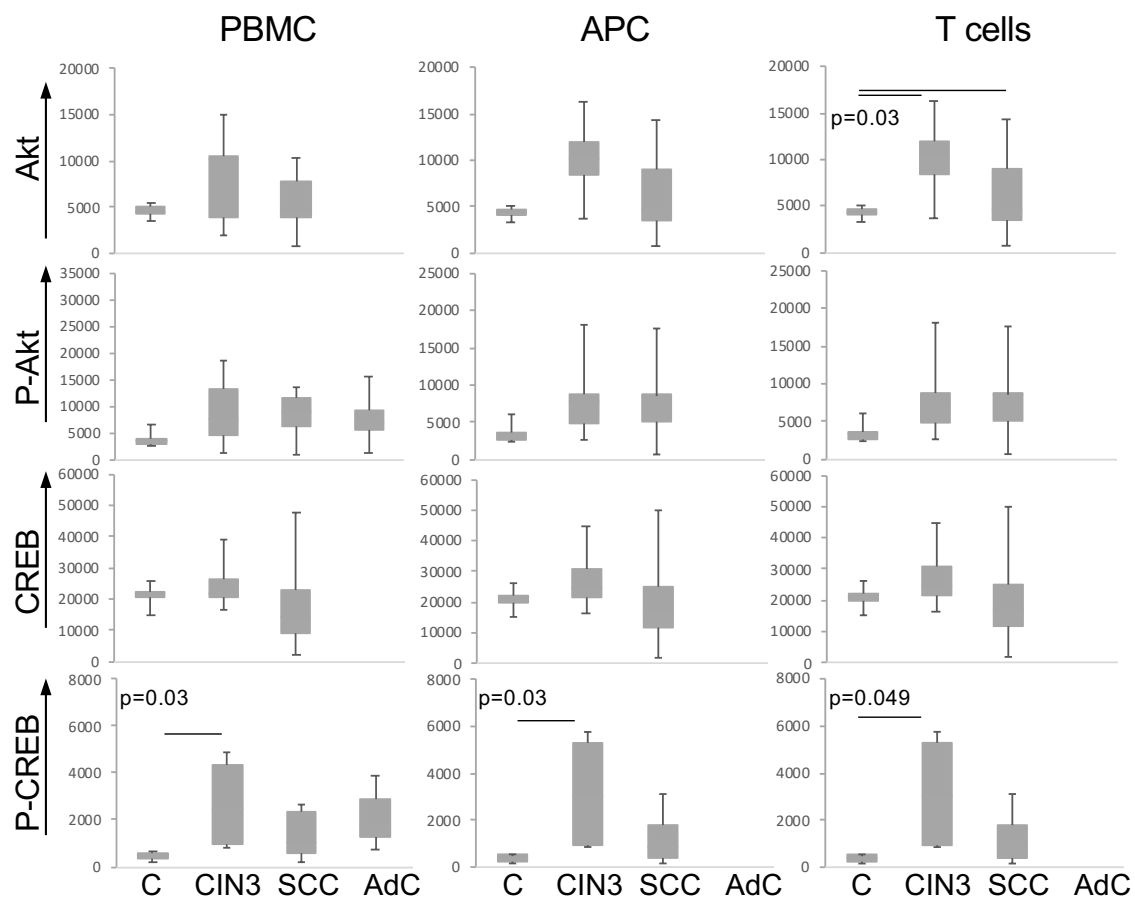

Rossetti et al. 2020  
Supplementary Figure 3

Supplement: Supplementary Figure 3 — Akt and CREB expression in circulating leukocyte populations according to lesion grade. PBMCs were labeled with anti-CD3 and anti-HLA-DR, then fixed and permeabilized and intracellularly stained with antibodies against the indicated proteins. Cells were analyzed in a FACSCanto, where, at least 10,000 events were acquired per sample. Data is represented as boxplots using the MFI value of each sample minus the background signal obtained with a sample incubated only with the secondary antibody. Experimental groups are: C – healthy donor controls, CIN3 – patients with high-grade lesions, SCC and AdC – patients with squamous cell carcinoma and adenocarcinomas. Bars and corresponding p values indicate significant differences between experimental groups. Sample sizes for Akt: CIN3 n=9, SCC=10, AdC=4; for CREB: CIN3 n=7, SCC=10, AdC=3. The samples sized for the AdC group were considered insufficient for analyzes. In all cases, we had 11 health donors. [file DataSheet_3.pdf]

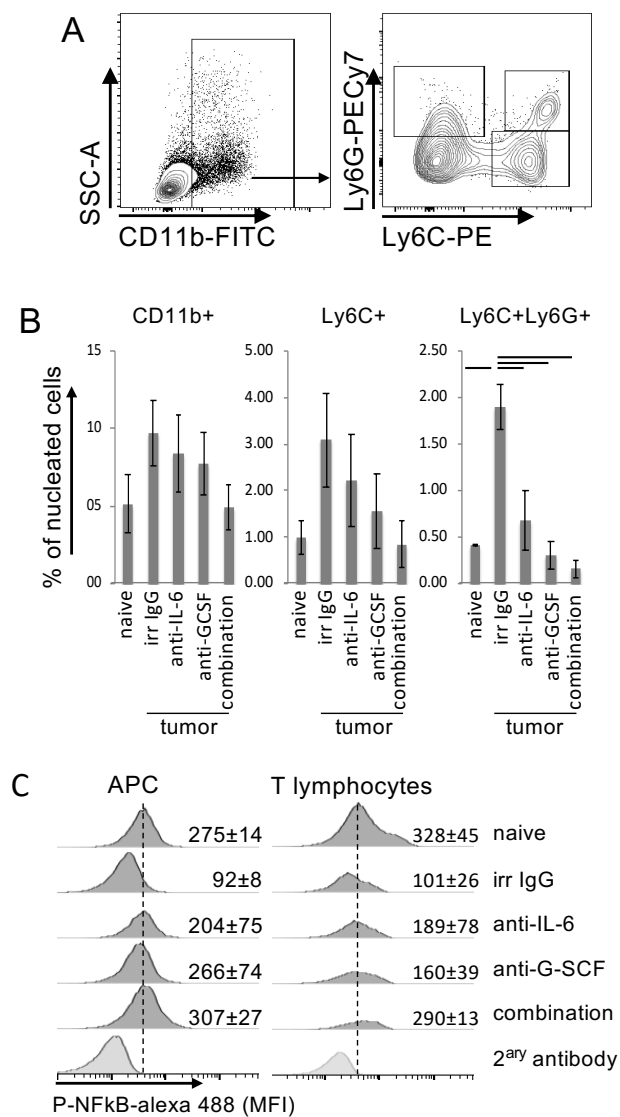

Rossetti et al. 2020  
Supplementary Figure 4

Supplement: Supplementary Figure 4 — Systemic effects of IL-6 and G-CSF neutralization. Spleens were harvested from the mice described in Figure 4 . Naïve mice and irrelevant IgG treated tumor-bearing mice, when applicable, were used as controls (naive and irr IgG, respectively). Splenocytes were labeled with antibodies and analyzed by flow cytometry to determine the frequency of leukocytes (A and B) and expression of phosphorylated p65-NFκB, after fixation and permeabilization (C). A. Gating strategy to identify spleen populations. To identify the myeloid populations first, we gated in the CD11b+ population, and within this population, we analyzed the Ly6C and Ly6G subpopulations. B. Frequency of splenocytes in naïve or tumor-bearing mice treated with the indicated antibodies. Average of 9 different animals per group is represented in the histograms. C. Phosphorylated-p65 NFκB expression measured by flow cytometry. Splenocytes labeled with anti-MHC-II (APC) and anti-CD3 (T cells), were fixed, permeabilized, and labeled with antibodies against phosphorylated-p65 NFκB. Histograms represent the median fluorescence intensity (MFI). Values indicated on the right side of histograms are the average and standard deviation of the MFI results from 3 mice. The indicated values are the signal obtained minus the MFI values of the unstained control. [file DataSheet_4.pdf]

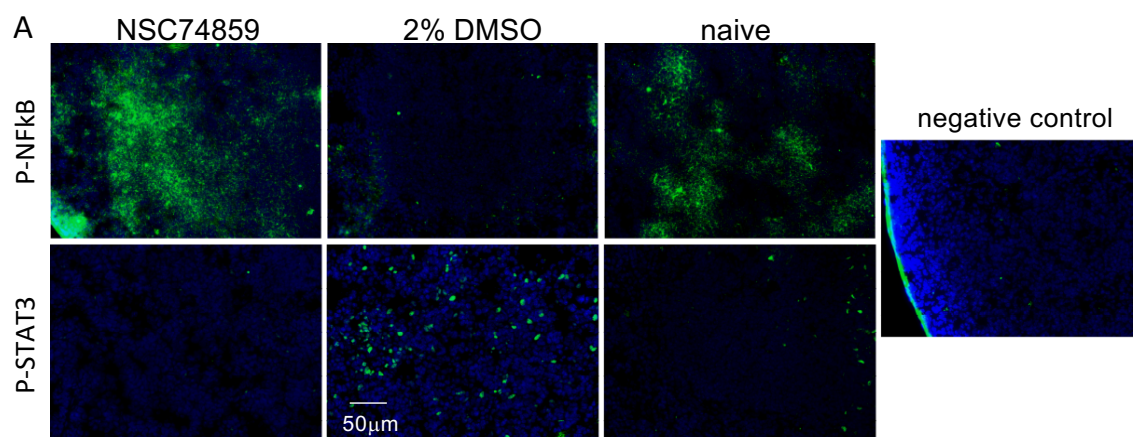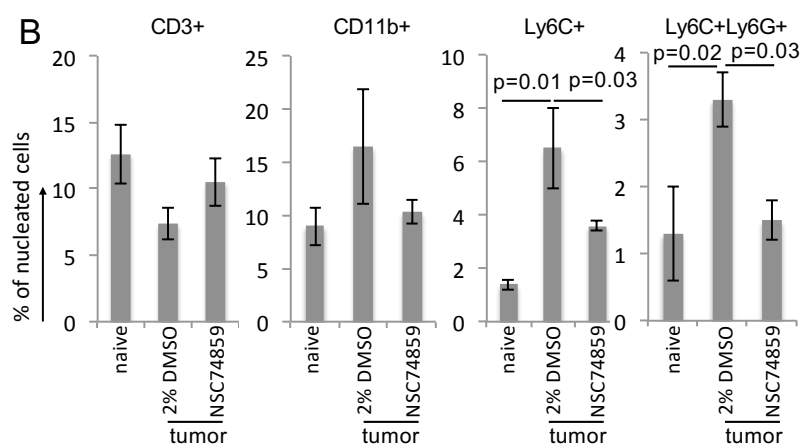

Supplement: Supplementary Figure 5 — Systemic effects of STAT3 inhibition. A. Phosphorylated STAT3 and p65 NFκB expression in spleen cryosections from different experimental groups (as described in Figure 6 ): – tumor-bearing mice treated with STAT3 inhibitor (NSC748590), tumor-bearing mice treated with 2%, and naïve – control mice without tumor cell inoculation. The negative control is a spleen section incubated only with Alexa 488 conjugated anti-rabbit antibody. B. Frequency of populations in the spleen of the indicated experimental groups. Splenocytes were labeled with antibodies against the indicated cell surface antigens and analyzed by flow cytometry. The histograms indicated the average of each population frequency in the different experimental groups. * indicate significant differences between the control tumor-bearing mice and naïve mice or mice treated with STAT3 inhibitor. [file DataSheet_5.pdf]
